# Supplementary material for: Men in menopause? Experimental verification of the mate choice theory with Drosophila melanogaster shows both sexes can undergo menopause
Source: PLoS One. 2025 Jul 3;20(7):e0326972. doi: 10.1371/journal.pone.0326972 (PMC12225806; doi:10.1371/journal.pone.0326972)
Supplement: S1 Table — (PDF) [file pone.0326972.s001.pdf]

Experimental Group	Replicate	Vial ID	Eggs Laid by Day (Generation						
			Day 1	Day 2	Day 3	Day 4	Day 5	Day 6	Day 7
Old male-young female	1M	1M_1	61	55	51	39	45	39	34
		1M_2	64	65	45	40	44	46	32
		1M_3	50	53	41	41	43	41	29
		1M_4	54	52	43	40	42	35	46
		1M_5	60	50	41	41	41	43	33
		1M_6	57	65	47	40	42	42	30
		1M_7	48	55	47	39	39	47	32
		1M_8	52	51	X	X	X	X	X
		1M_9	64	58	45	38	47	43	38
		1M_10	68	51	51	41	40	40	30
	2M	2M_1	59	52	45	57	41	42	33
		2M_2	51	52	44	37	39	46	34
		2M_3	63	50	45	40	38	42	32
		2M_4	66	57	43	40	45	30	32
		2M_5	55	62	46	38	39	45	42
		2M_6	60	52	49	37	46	41	34
		2M_7	49	61	45	38	42	32	33
		2M_8	65	51	44	39	39	31	30
		2M_9	56	55	46	38	40	45	43
		2M_10	62	54	41	41	39	34	29
		2M_11	71	49	39	31	43	38	31
		2M_12	59	60	38	36	49	40	36
	3M	3M_1	65	62	58	47	39	44	51
		3M_2	60	66	61	40	44	43	40
		3M_3	61	60	66	44	40	36	53
		3M_4	65	67	64	44	43	42	50
		3M_5	65	51	64	43	43	45	42
		3M_6	63	68	60	50	51	45	44
		3M_7	62	63	65	44	40	30	44
		3M_8	68	67	59	46	49	47	52
		3M_9	61	61	57	45	50	43	51
		3M_10	62	59	59	43	45	54	46
		3M_11	57	67	63	47	50	43	49
		3M_12	65	60	63	42	40	48	42
		1F_1	60	69	50	54	61	48	41
		1F_2	66	59	49	64	60	50	49
		1F_3	65	66	62	56	57	51	54
		1F_4	64	68	60	59	60	47	45

Old female-young male	1F	1F_5	61	63	56	63	62	55	58
		1F_6	57	56	60	60	57	58	48
		1F_7	54	58	65	64	58	47	55
		1F_8	55	57	63	63	62	51	49
		1F_9	54	54	56	62	58	58	48
		1F_10	54	54	64	61	59	52	60
	2F	2F_1	63	59	70	63	57	51	46
		2F_2	58	68	66	56	50	52	44
		2F_3	65	68	63	62	62	57	54
		2F_4	68	57	57	60	56	53	53
		2F_5	62	65	59	57	60	48	49
		2F_6	57	66	63	57	64	52	51
		2F_7	57	61	69	62	63	55	50
		2F_8	68	47	65	61	58	52	48
		2F_9	66	65	60	55	64	50	53
		2F_10	68	61	62	63	57	49	45
	3F	3F_1	60	59	50	43	50	46	45
		3F_2	59	47	57	45	46	31	33
		3F_3	54	59	49	54	46	52	35
		3F_4	55	50	55	50	43	50	34
		3F_5	56	60	57	49	42	47	40
		3F_6	56	49	52	46	51	53	49
		3F_7	55	48	53	43	53	49	49
		3F_8	50	49	57	40	54	38	45
		3F_9	56	53	60	58	41	38	44
		3F_10	48	54	57	56	52	52	39
Control	C	C_1	65	66	65	67	67	60	64
		C_2	69	73	72	64	64	X	X
		C_3	74	75	67	68	66	62	58
		C_4	75	74	64	71	69	63	55
		C_5	67	65	64	72	65	57	64
		C_6	65	72	72	71	67	64	61
		C_7	66	71	65	70	56	63	63
		C_8	68	69	75	66	65	60	62
		C_9	73	70	72	65	71	55	57
		C_10	68	74	73	67	72	61	57
		C_11	73	74	69	67	69	59	64
		C_12	72	70	73	78	73	55	57
		C_13	75	73	72	70	67	56	60
		C_14	70	70	73	69	66	58	57
		C_15	71	74	72	63	72	55	58

		C_16	68	75	72	60	70	62	62
		C_17	70	68	67	X	X	X	X
		C_18	62	74	70	71	68	63	60
		C_19	76	71	73	66	68	61	57
		C_20	60	71	67	69	73	55	55

NOTE: "X" indicates the fly died.

n 20) in Each Experimental Group

Day 8	Day 9	Day 10	Day 11	Day 12	Day 13	Day 14	Day 15	Day 16	Day 17	Day 18	Day 19
30	26	33	31	28	19	28	18	21	23	11	16
29	33	29	26	30	24	15	18	19	11	18	10
34	33	34	34	25	15	21	24	18	20	17	16
32	28	17	26	15	30	23	17	11	15	18	17
31	27	33	27	29	26	15	19	17	14	11	9
32	31	28	32	28	29	20	15	16	19	14	8
32	34	31	27	22	21	23	16	10	10	16	18
X	X	X	X	X	X	X	X	X	X	X	X
33	26	22	27	22	20	25	22	12	15	17	13
30	33	36	34	X	X	X	X	X	X	X	X
30	X	X	X	X	X	X	X	X	X	X	X
32	30	27	26	26	20	19	22	21	12	17	8
30	X	X	X	X	X	X	X	X	X	X	X
34	29	30	31	27	14	15	21	16	17	18	12
32	28	26	32	25	26	15	22	18	10	15	7
30	31	29	32	16	31	20	17	13	17	12	10
33	32	30	28	26	30	23	18	10	21	10	18
33	33	26	27	30	23	16	21	11	10	18	13
29	29	28	26	30	29	17	22	17	15	10	8
42	34	21	39	25	31	25	23	14	18	8	15
33	31	31	29	21	22	17	25	16	20	11	15
42	25	27	30	22	27	18	23	20	19	11	15
32	36	32	39	25	36	32	22	29	21	22	16
35	36	44	33	30	32	26	24	27	16	12	14
42	40	34	40	30	30	27	29	24	5	17	18
33	44	44	35	31	32	27	28	22	6	10	9
35	35	35	37	33	36	26	27	25	19	X	X
36	34	28	31	35	27	30	27	28	13	9	11
31	34	44	34	32	32	22	30	22	13	20	14
43	43	31	39	X	X	X	X	X	X	X	X
33	40	42	31	30	35	22	29	22	13	17	16
30	33	30	30	35	29	29	30	29	17	20	11
54	X	X	X	X	X	X	X	X	X	X	X
39	42	32	31	35	35	25	32	25	8	11	17
37	24	15	15	19	21	23	X	X	X	X	X
44	31	29	29	15	19	14	24	19	14	15	9
39	29	26	26	20	26	16	21	17	11	19	14
48	25	25	25	16	14	18	19	14	10	12	11

46	26	22	22	19	14	16	21	17	8	15	10
42	29	27	27	27	23	24	16	19	10	10	9
41	22	34	34	21	26	21	21	9	12	15	11
48	34	26	26	15	21	24	14	20	11	15	8
43	31	23	23	24	14	15	21	18	15	14	12
42	24	26	26	28	25	18	20	19	13	18	17
44	35	33	27	28	28	23	20	21	14	0	0
51	36	32	17	28	26	25	13	16	13	10	11
45	28	31	33	26	35	23	11	17	9	19	5
48	27	33	27	20	20	20	18	17	10	12	11
45	29	27	26	20	24	15	24	14	15	12	14
46	28	31	25	34	22	16	14	13	16	10	0
47	35	32	26	23	21	20	14	18	12	15	11
46	31	35	31	34	23	21	20	15	11	0	0
54	30	28	28	33	33	11	19	14	15	14	13
54	30	33	27	26	21	22	18	11	20	17	11
41	25	51	30	31	15	15	28	15	24	14	21
43	52	28	21	30	17	21	10	15	19	19	16
43	44	29	23	22	16	16	17	13	21	17	17
43	31	20	30	25	19	17	15	17	10	11	8
48	46	41	29	14	24	14	12	17	20	18	17
46	37	33	26	X	X	X	X	X	X	X	X
48	33	21	31	37	13	22	14	13	20	13	11
35	30	37	26	38	22	19	25	14	20	21	21
35	36	46	30	30	26	16	15	18	21	10	13
48	54	30	17	39	17	19	10	10	17	18	10
55	59	52	42	38	35	41	29	17	10	6	0
X	X	X	X	X	X	X	X	X	X	X	X
63	60	53	36	44	36	33	32	21	23	15	16
63	57	51	42	41	34	47	34	17	19	10	29
62	64	53	44	38	33	33	32	17	18	13	21
58	55	48	39	43	39	33	18	18	17	14	21
61	57	51	46	37	48	33	33	20	22	10	11
60	56	51	42	43	45	44	29	22	17	14	13
64	60	59	47	38	32	34	35	20	19	27	24
59	58	53	46	46	45	33	30	22	21	14	10
55	64	49	40	38	45	39	34	22	22	7	9
56	61	49	46	38	38	39	23	18	18	10	14
57	61	48	59	40	33	38	39	18	19	17	17
56	42	55	39	37	42	32	21	20	20	15	9
60	55	55	43	45	44	45	37	19	19	11	13

49	56	54	43	42	47	35	38	20	21	10	13
X	X	X	X	X	X	X	X	X	X	X	X
60	61	50	44	40	34	48	39	19	19	15	14
63	57	52	36	45	42	45	38	18	19	11	17
61	61	54	38	36	34	47	30	20	17	9	13

	Offspring Hatched Over 3 Day Period					
Day 20	Day 3	Day 6	Day 9	Day 12	Day 15	Day 18
13	28	39	28	16	0	0
11	49	43	21	33	0	0
12	35	41	24	0	0	0
15	35	44	34	0	0	0
14	32	34	37	19	0	0
8	21	38	17	0	0	0
14	22	32	22	20	12	27
X	X	X	X	X	X	X
8	34	44	16	0	0	0
X	24	32	24	X	X	X
X	33	43	X	X	X	X
9	40	33	0	0	0	0
X	36	45	X	X	X	X
7	49	37	27	23	0	0
6	32	6	42	26	23	2
12	44	45	38	23	0	0
11	4	14	40	37	21	23
14	46	19	0	0	0	0
7	12	7	42	15	0	0
14	14	14	0	0	0	0
14	32	20	32	44	39	11
14	52	17	47	25	1	0
18	49	47	51	22	0	0
12	20	29	0	0	0	0
9	48	53	48	28	11	0
14	37	4	1	0	0	0
X	32	19	40	28	38	X
16	55	47	49	13	0	0
11	46	27	38	19	28	8
X	38	38	15	X	X	X
18	36	9	44	0	0	0
11	15	10	0	0	0	0
X	17	15	X	X	X	X
19	36	25	40	27	35	20
X	28	24	42	26	X	X
13	42	23	46	31	15	6
5	40	22	44	34	23	11
12	35	18	48	22	12	13

11	46	22	43	29	20	0
10	41	26	48	37	13	0
7	43	28	32	23	0	0
15	38	34	36	41	18	14
12	34	22	34	39	21	15
8	40	23	20	32	19	0
0	42	28	29	16	0	0
0	43	26	18	0	0	0
5	37	28	25	0	0	0
0	45	20	22	22	0	0
12	35	23	16	0	0	0
0	38	24	22	0	0	0
0	36	29	30	15	0	0
0	39	21	11	0	0	0
16	41	29	27	20	0	0
11	34	19	12	0	0	0
14	43	41	48	31	0	0
10	39	39	58	9	0	0
0	37	37	45	35	0	0
0	45	35	37	39	0	0
11	34	37	23	28	0	0
X	36	41	11	X	X	X
10	32	45	24	29	6	0
14	37	40	41	0	0	0
14	47	39	32	21	0	0
11	26	35	0	0	0	0
0	63	57	42	22	18	4
X	65	52	50	34	28	7
13	49	56	49	19	30	7
14	55	49	44	37	15	10
20	51	50	49	40	21	17
15	64	54	46	29	25	0
18	50	X	X	X	X	X
11	58	57	47	32	17	11
20	57	53	42	27	19	5
14	63	59	50	40	21	4
10	61	57	50	32	14	8
16	54	57	46	38	24	9
16	66	56	45	29	30	3
21	59	61	41	22	17	10
14	58	51	43	26	25	13

18	69	58	50	31	21	6
X	57	X	X	X	X	X
20	59	58	42	30	21	5
19	48	56	45	27	12	2
20	62	59	50	34	16	0
