# Supplementary material for: Men in menopause? Experimental verification of the mate choice theory with Drosophila melanogaster shows both sexes can undergo menopause
Source: PLoS One. 2025 Jul 3;20(7):e0326972. doi: 10.1371/journal.pone.0326972 (PMC12225806; doi:10.1371/journal.pone.0326972)
Supplement: S2 Table — (PDF) [file pone.0326972.s002.pdf]

Experimental Group	Replicate	Vial ID	Day 1	Day 2	Day 3	Day 4	Day 5	Day 6	Day 7
Old male-young female	1M	1M_1	52	54	41	17	21	19	15
		1M_2	53	41	39	43	19	13	11
		1M_3	49	43	29	17	18	18	13
		1M_4	47	39	43	28	23	16	14
		1M_5	33	36	46	38	31	23	23
		1M_6	48	51	42	36	21	16	16
		1M_7	42	38	31	37	14	18	11
		1M_8	36	39	27	30	22	19	14
		1M_9	36	43	29	40	26	19	16
		1M_10	43	39	47	X	X	X	X
	2M	2M_1	42	40	46	37	22	17	12
		2M_2	37	29	35	33	17	16	13
		2M_3	43	36	39	36	14	17	11
		2M_4	39	37	36	43	20	18	18
		2M_5	39	37	37	44	21	20	17
		2M_6	38	34	26	19	18	19	12
		2M_7	48	43	29	23	14	14	13
		2M_8	39	40	39	27	20	19	19
		2M_9	52	43	48	23	19	19	15
		2M_10	51	47	39	29	23	19	15
	3M	3M_1	51	48	62	26	23	21	21
		3M_2	49	38	47	30	29	29	21
		3M_3	45	41	38	36	38	26	21
		3M_4	48	41	38	35	27	23	22
		3M_5	41	48	47	39	29	21	19
		3M_6	38	35	39	23	21	19	19
		3M_7	43	49	37	31	29	20	17
		3M_8	42	47	38	29	21	21	19
		3M_9	46	49	36	32	39	29	16
		3M_10	41	46	39	32	17	28	19
		C_1	78	58	61	59	54	X	X
		C_2	69	49	64	59	57	33	31
		C_3	67	60	65	71	69	37	36
		C_4	77	X	X	X	X	X	X
		C_5	65	59	48	46	47	37	33
		C_6	63	52	71	67	63	41	39
		C_7	71	72	71	77	73	46	41
		C_8	69	61	89	76	75	30	28

**Control**	**Control**	C_9	73	58	49	41	39	37	21
		C_10	88	85	49	49	47	45	51
		C_11	71	87	61	59	57	51	38
		C_12	62	64	68	64	61	37	33
		C_13	61	59	58	48	43	38	25
		C_14	68	62	89	76	74	39	39
		C_15	73	69	71	68	69	66	30
		C_16	71	61	52	51	49	47	36
		C_17	77	62	70	65	61	41	36
		C_18	83	76	78	63	60	40	39
		C_19	64	53	62	59	55	49	33
		C_20	61	59	58	57	53	46	35

NOTE: X is used if a fly dies.

NOTE: Only data for Old male-young female and control lines were collected for fertility a

Experimental group	Replicate	Time	Day 0	Day 3	Day 6	Day 9	Day 12	Day 15	Day 18
**Control**	**Control**	# survivors	150	150	147	129	127	121	120
		# lost	0	0	5	0	1	0	0
		# at risk	150	150	144.5	129	126.5	121	120
		# events	0	3	13	2	5	1	3
**Old male-young female**	**1M**	# survivors	50	50	50	44	42	36	32
		# lost	0	0	0	0	2	0	0
		# at risk	50	50	50	44	41	36	32
		# events	0	0	6	2	4	4	6
	**2M**	# survivors	50	50	50	48	48	47	47
		# lost	0	0	0	0	0	0	0
		# at risk	50	50	50	48	48	47	47
		# events	0	0	2	0	1	0	0
	**3M**	# survivors	50	50	50	50	48	48	47
		# lost	0	0	0	0	0	0	0
		# at risk	50	50	50	50	48	48	47
		# events	0	0	0	2	0	1	0
**Old female-**	**1F**	# survivors	50	50	49	43	43	43	43
		# lost	0	0	2	0	0	0	0
		# at risk	50	50	48	43	43	43	43
		# events	0	1	4	0	0	0	0
	**2F**	# survivors	50	50	49	46	41	41	40
		# lost	0	0	0	2	0	0	0

young male	2F	# at risk	50	50	49	45	41	41	40
		# events	0	1	3	3	0	1	2
	3F	# survivors	50	50	50	50	50	50	50
		# lost	0	0	0	0	0	0	0
		# at risk	50	50	50	50	50	50	50
		# events	0	0	0	0	0	0	1

Experimental group	Group	Time	Day 0	Day 3	Day 6	Day 9	Day 12	Day 15	Day 18
Control	Control	# survivors	150	150	145	136	134	132	130
		# lost	0	0	2	0	0	0	3
		# at risk	150	150	144	136	134	132	128.5
		# events	0	5	7	2	2	2	6
Old male-young female	1M	# survivors	50	50	45	41	40	33	30
		# lost	0	2	0	0	2	0	0
		# at risk	50	49	45	41	39	33	30
		# events	0	3	4	1	5	3	2
	2M	# survivors	50	50	49	48	48	47	46
		# lost	0	0	0	0	0	0	0
		# at risk	50	50	49	48	48	47	46
		# events	0	1	1	0	1	1	4
	3M	# survivors	50	50	47	46	45	41	40
		# lost	0	1	0	0	1	0	0
		# at risk	50	49.5	47	46	44.5	41	40
		# events	0	2	1	1	3	1	2
Old female-young male	1F	# survivors	50	50	50	48	48	40	40
		# lost	0	0	0	0	2	0	0
		# at risk	50	50	50	48	47	40	40
		# events	0	0	2	0	6	0	5
	2F	# survivors	50	50	50	49	48	47	47
		# lost	0	0	0	0	0	0	3
		# at risk	50	50	50	49	48	47	45.5
		# events	0	0	1	1	1	0	5
	3F	# survivors	50	50	50	45	41	34	34
		# lost	0	0	1	0	1	0	0
		# at risk	50	50	49.5	45	40.5	34	34
		# events	0	0	4	4	6	0	0

Ovariole and Stage-14 Egg C									
			Stage-14 Egg Chamber Counts (average of b						
Experimental Group	Replicate	Sample #	Day 3	Day 6	Day 9	Day 12	Day 15	Day 18	Day 21
Old male-young female	1M	1	8.5	5	4	6	2	3.5	3
		2	6.5	2.5	5	4.5	1.5	3	2.5
		3	8	5.5	4.5	3	1.5	3.5	2.5
	2M	1	7.5	6	0.5	3	1.5	3	3
		2	7.5	5.5	7	4.5	2	6.5	3
		3	6.5	1	4.5	4	2.5	5	2.5
	3M	1	9	5	5.5	4.5	1.5	6.5	3.5
		2	4.5	2.5	4.5	5.5	1	3	2.5
		3	4.5	3	4.5	4	1.5	4	4.5
Old female-young male	1F	1	10.5	16	14	10	0	8.5	3.5
		2	9	10.5	10	11	10	9	2.5
		3	10.5	9.5	9.5	9.5	6.5	6.5	2.5
	2F	1	9.5	3.5	14.5	12.5	9.5	8	4
		2	9.5	9	9.5	12	7	6.5	4
		3	10.5	6.5	12	9.5	5.5	7.5	3.5
	3F	1	9	8.5	9	12.5	5	5.5	1.5
		2	12	3	9	12	5.5	8.5	5.5
		3	10.5	4.5	12	8.5	4	7	3.5
Control	Control	1	11.5	8.5	10.5	9.5	7	9.5	8.5
		2	12.5	8.5	11	14	6	9.5	8.5

	Length an								
Experimental Group	Replicate	Sample #	Day 0		Day 5		Day 10		Day
			Length	Width	Length	Width	Length	Width	Length
Old male-young female	1M	1	2.7	0.16	2.52	0.19	2.21	0.135	2.4
		2	1.89	0.25	2.52	0.155	1.91	0.145	2
		3	1.71	0.165	2.1	0.14	2.25	0.11	2.16
		4	1.96	0.22	2.11	0.165	2.18	0.1	2
		5	1.85	0.215	2.16	0.155	1.87	0.115	2.01
	2M	1	1.86	0.22	1.74	0.14	2.02	0.135	2.2
		2	1.72	0.21	1.865	0.11	1.5	0.1	2.015
		3	1.725	0.18	2.02	0.135	2.02	0.12	1.88
		4	1.625	0.165	1.75	0.16	2.18	0.12	2.06
		5	2	0.15	1.75	0.15	2.04	0.12	2.01

	**3M**	**1**	2.09	0.18	2.7	0.1	1.93	0.14	2.2
		**2**	2.44	0.175	1.75	0.145	2.06	0.13	1.76
		**3**	1.61	0.165	2.4	0.16	2.12	0.16	2.15
		**4**	1.98	0.17	2.415	0.125	1.91	0.115	1.87
		**5**	2.255	0.155			2.02	0.105	1.87
**Old female-young male**	**1F**	**1**	2.18	0.18	1.84	0.125	2.06	0.095	2.085
		**2**	2.075	0.18	1.985	0.105	1.76	0.135	2.1
		**3**	1.78	0.1	1.885	0.135	2.055	0.115	2.35
		**4**	1.935	0.19	1.905	0.1	1.5	0.12	2.09
		**5**	1.785	0.16	1.84	0.13	1.78	0.1	2.06
	**2F**	**1**	1.64	0.135	1.695	0.165	1.84	0.12	1.87
		**2**	1.895	0.14	2.265	0.095	1.75	0.09	1.89
		**3**	2.055	0.16	2.325	0.1	1.58	0.065	1.825
		**4**	2.21	0.125	1.945	0.095	1.86	0.12	2.55
		**5**	1.76	0.19	2.105	0.125	1.81	0.11	1.78
	**3F**	**1**	1.42	0.16	1.765	0.15	2.295	0.1	2.65
		**2**	1.66	0.1	1.9	0.19	1.87	0.1	2.21
		**3**	1.8	0.13	1.705	0.105	2.025	0.135	1.71
		**4**	1.585	0.145	1.92	0.11	1.86	0.1	1.845
		**5**	1.64	0.11	2.01	0.12	1.69	0.105	1.8
**Control**	**Control**	**1**	1.88	0.125	2.2	0.12	1.79	0.145	2
		**2**	1.94	0.16	1.815	0.11	1.83	0.14	1.775
		**3**	1.95	0.16	2.01	0.14	1.9	0.12	1.81
		**4**	2.17	0.24	2.16	0.13	1.855	0.125	2.125
		**5**	1.84	0.225	1.83	0.13	1.89	0.13	1.73

Number of Eggs Laid per Day											
Day 8	Day 9	Day 10	Day 11	Day 12	Day 13	Day 14	Day 15	Day 16	Day 17	Day 18	Day 19
15	17	14	10	9	0	9	7	7	5	5	2
13	14	16	17	6	9	5	5	8	3	2	0
14	15	15	14	11	9	9	8	9	3	2	2
14	11	16	13	11	X	X	X	X	X	X	X
19	17	16	16	13	14	11	10	10	6	4	0
13	14	16	11	11	10	6	8	8	3	3	1
15	16	11	11	7	9	6	5	5	4	6	3
14	13	9	11	11	9	9	7	9	5	4	2
14	14	15	15	13	13	11	9	5	7	4	4
X	X	X	X	X	X	X	X	X	X	X	X
11	10	9	10	7	7	5	5	6	4	3	3
13	9	9	10	6	6	5	4	8	2	2	1
14	9	7	6	5	5	4	3	8	2	2	1
17	16	13	12	12	11	11	9	8	5	5	3
17	15	11	10	7	7	8	8	5	5	3	2
18	11	11	11	9	9	7	7	5	4	7	1
13	12	12	11	9	9	7	5	7	3	2	2
16	15	14	14	13	13	11	9	9	5	4	4
19	13	12	10	6	9	8	8	8	5	4	2
15	12	12	11	10	10	9	7	7	5	5	4
19	17	13	15	10	13	18	11	9	7	9	4
17	15	13	15	11	11	9	9	6	6	4	2
18	15	16	18	13	13	11	9	10	9	7	3
17	14	13	14	9	11	9	9	8	7	9	5
19	17	19	19	13	13	11	12	9	5	7	4
17	16	19	14	12	15	14	14	11	9	8	8
X	X	X	X	X	X	X	X	X	X	X	X
18	18	17	16	16	15	13	15	12	9	8	7
18	17	19	15	7	13	13	11	9	12	7	3
19	16	16	15	9	14	11	10	10	9	9	5
X	X	X	X	X	X	X	X	X	X	X	X
27	21	19	15	15	14	12	12	11	11	10	9
35	33	29	21	14	16	15	15	13	11	11	10
X	X	X	X	X	X	X	X	X	X	X	X
31	31	27	18	15	13	13	14	12	9	11	10
37	28	21	17	13	18	13	13	11	8	8	7
39	37	28	21	17	15	15	13	9	8	8	7
26	26	21	19	15	14	12	12	11	11	9	8

19	19	29	12	12	11	11	9	7	5	6	6
38	31	39	19	17	21	13	13	11	9	9	11
38	35	41	22	19	13	13	17	12	11	11	10
29	21	23	19	17	15	13	16	11	10	10	7
37	23	19	15	14	12	12	8	11	8	8	5
36	29	19	19	17	15	15	14	12	12	8	4
28	21	28	17	17	19	11	15	13	11	9	5
39	27	23	18	13	15	14	11	11	9	6	6
36	29	31	23	19	21	18	16	14	15	8	6
30	25	29	22	20	16	12	18	15	13	9	9
28	15	17	19	17	14	10	19	13	9	6	8
22	19	19	13	16	13	10	13	11	7	8	7

ınd fecundity data.

				FEMALE Fly Survival, Deaths, and Numbers at Risk by Tim							
**Day 21**	**Day 24**	**Day 27**	**Day 30**	**Day 33**	**Day 36**	**Day 39**	**Day 42**	**Day 45**	**Day 48**	**Day 51**	**Day 54**
117	115	113	110	106	105	99	97	92	87	78	75
0	0	0	0	1	0	0	0	0	2	0	2
117	115	113	110	105.5	105	99	97	92	86	78	74
2	2	3	4	0	6	2	5	5	7	3	9
26	21	14	11	6	5	3	0	0	0	0	0
0	2	0	1	0	1	0	0	0	0	0	0
26	20	14	10.5	6	4.5	3	0	0	0	0	0
5	5	3	4	1	1	3	0	0	0	0	0
47	46	43	40	39	38	37	37	35	35	34	28
0	0	1	0	0	0	0	0	0	0	2	0
47	46	42.5	40	39	38	37	37	35	35	33	28
1	3	2	1	1	1	0	2	0	1	4	3
47	46	44	41	35	30	23	22	19	19	18	16
0	0	1	3	0	3	0	0	0	0	0	0
47	46	43.5	39.5	35	28.5	23	22	19	19	18	16
1	2	2	3	5	4	1	3	0	1	2	2
43	43	43	40	40	37	35	32	28	25	24	21
0	0	0	0	0	0	0	1	0	0	0	1
43	43	43	40	40	37	35	31.5	28	25	24	20.5
0	0	3	0	3	2	3	3	3	1	3	4
38	35	32	29	23	23	19	17	14	13	13	11
0	0	0	2	0	0	0	1	0	0	0	0

38	35	32	28	23	23	19	16.5	14	13	13	11
3	3	3	4	0	4	2	2	1	0	2	2
49	49	48	45	45	44	43	43	39	38	35	33
0	0	0	0	0	0	0	1	0	0	0	0
49	49	48	45	45	44	43	42.5	39	38	35	33
0	1	3	0	1	1	0	3	1	3	2	0

MALE Fly Survival, Deaths, and Numbers at Risk by Time (in Days)

Day 21	Day 24	Day 27	Day 30	Day 33	Day 36	Day 39	Day 42	Day 45	Day 48	Day 51	Day 54
121	113	90	76	67	57	48	47	41	36	29	25
0	6	1	1	2	0	0	1	0	0	0	1
121	110	89.5	75.5	66	57	48	46.5	41	36	29	24.5
8	17	13	8	8	9	1	5	5	7	4	2
28	22	20	16	12	11	10	8	6	3	0	0
2	0	0	1	0	0	0	0	0	1	0	0
27	22	20	15.5	12	11	10	8	6	2.5	0	0
4	2	4	3	1	1	2	2	3	2	0	0
42	41	40	39	36	34	32	28	25	25	18	16
0	0	0	1	0	0	0	0	0	2	0	0
42	41	40	38.5	36	34	32	28	25	24	18	16
1	1	1	2	2	2	4	3	0	5	2	5
38	33	32	29	27	24	19	14	8	4	0	0
0	0	0	0	0	0	1	2	0	1	0	0
38	33	32	29	27	24	18.5	13	8	3.5	0	0
5	1	3	2	3	5	4	4	4	3	0	0
35	31	31	25	24	19	15	15	11	10	10	8
0	0	3	0	0	0	0	0	0	0	0	1
35	31	29.5	25	24	19	15	15	11	10	10	7.5
4	0	3	1	5	4	0	4	1	0	2	2
39	32	25	23	21	16	16	12	12	10	8	6
0	2	0	0	0	0	1	0	0	0	0	0
39	31	25	23	21	16	15.5	12	12	10	8	6
7	5	2	2	5	0	3	0	2	2	2	2
34	32	31	30	22	21	19	16	14	11	9	4
0	0	0	2	0	0	0	0	0	0	2	0
34	32	31	29	22	21	19	16	14	11	8	4
2	1	1	6	1	2	3	2	3	2	3	2

hamber Counts of Female Flies

oth sides)			Ovariole Counts (average of both sides)								
Day 24	Day 27	Day 30	Day 3	Day 6	Day 9	Day 12	Day 15	Day 18	Day 21	Day 24	Day 27
2.5	1.5	0.5	13.5	16	11	14.5	11.5	12	12	13.5	10.5
5	2	1.5	12	15.5	11.5	12.5	12	13	9.5	13.5	11
4.5	2	1	13	15	10.5	14.5	11.5	13	10	12	10
8	2.5	0.5	16	16	16	14.5	13.5	19.5	10.5	13.5	9.5
7	1.5	0.5	13	15.5	17.5	15.5	15.5	18	14.5	13.5	10.5
6.5	2	1	14.5	16.5	16.5	14	14.5	18.5	14.5	13	11
2.5	1	1.5	14	13	11	12	9.5	14.5	15.5	13	9.5
4.5	1	0.5	12	15.5	10	12.5	14	10	15	13.5	9.5
2.5	1.5	0.5	13.5	17.5	11	15.5	13.5	9.5	14.5	12.5	10
7.5	3.5	1	14.5	16	16.5	13.5	14	19	11	15.5	10
8.5	3	1.5	14	14.5	15	14	17.5	18.5	11.5	17	13.5
8.5	2.5	1.5	15.5	14.5	14.5	13.5	14.5	17.5	13.5	15	10.5
9	3	1.5	14	14.5	17.5	12.5	16.5	17.5	13.5	15.5	9.5
10.5	4	1	12.5	15	18.5	14.5	17.5	17	14.5	14.5	9.5
8	3.5	1.5	13.5	14.5	15.5	13.5	17.5	17	13.5	17.5	10
9.5	4	0.5	12	15	15	13.5	17	17.5	10.5	16	10
5.5	3	0.5	13.5	17	14	12.5	16.5	17.5	12.5	15	10.5
8	2.5	1	13	17	15.5	12.5	14.5	17	13.5	15.5	13
9.5	5	2.5	14.5	17.5	15.5	15	18	15.5	15.5	15.5	12.5
9.5	4	1.5	14.5	16.5	17.5	14.5	18	15.5	16	16.5	12

d Width of Male Fly Testis per Day (in mm)

y 15	Day 20		Day 25		Day 30		Day 35		Day 40		Day
Width	Length	Width	Length	Width	Length	Width	Length	Width	Length	Width	Length
0.14	1.975	0.115	2.14	0.1	2.27	0.095	2.175	0.095	1.91	0.105	2.14
0.075	1.97	0.135	1.95	0.155	2.24	0.1	2.385	0.095	2.2	0.1	2.21
0.125	1.965	0.1	2.01	0.15	2.3	0.09	2.26	0.1	2.06	0.14	2.265
0.105	2.35	0.115	2.48	0.115	1.8	0.1	2.06	0.125	2.06	0.105	2.08
0.12	1.62	0.095	2.29	0.13	1.92	0.11	2.16	0.105	2.02	0.1	2.195
0.12	2	0.12	1.96	0.15	2.02	0.095	2.35	0.1	2.01	0.1	2.105
0.13	1.77	0.125	2.24	0.1	1.74	0.09	2.135	0.105	2.16	0.1	2.23
0.14	2.04	0.1	1.95	0.1	2.21	0.09	2.08	0.12	2.08	0.08	2.075
0.105	2.095	0.105	1.98	0.105	2.3	0.12	2.09	0.1	2.3	0.1	2.045
0.105	2.32	0.11	2	0.115	2.2	0.105	2.185	0.1	2.135	0.11	2.05

0.12	1.84	0.115	1.675	0.135	2.32	0.09	1.975	0.105	2.32	0.09	2.25
0.115	1.91	0.115	2.15	0.11	1.98	0.105	2.03	0.105	1.95	0.135	2.12
0.11	2.2	0.16	1.98	0.17	2.115	0.11	2.3	0.09	2.075	0.11	2.34
0.125	1.82	0.17	1.82	0.09	1.78	0.13	2.02	0.1	2.13	0.1	2.26
0.115	1.87	0.105	1.92	0.11	2.02	0.105	2	0.105	2.085	0.11	2.12
0.11	1.91	0.11	1.63	0.08	1.89	0.07	2.14	0.09	2.31	0.07	2.04
0.1	1.57	0.08	1.91	0.07	1.94	0.12	2.085	0.085	1.995	0.08	2.185
0.08	1.86	0.095	1.915	0.13	2.04	0.08	2.52	0.07	2.01	0.09	2.05
0.11	2.13	0.115	1.93	0.14	2	0.08	2.1	0.09	1.94	0.08	2.21
0.105	1.96	0.11	1.64	0.11	1.915	0.115	2.165	0.075	1.84	0.1	2
0.11	1.68	0.14	2.14	0.11	2.3	0.14	2.2	0.08	2.005	0.085	2.28
0.105	2.12	0.11	2.165	0.12	1.85	0.12	1.81	0.085	1.94	0.09	2.16
0.095	1.91	0.105	2.02	0.11	2.07	0.1	1.925	0.105	1.915	0.1	2.175
0.105	1.795	0.07	2.04	0.1	2.105	0.09	2.1	0.1	2.24	0.09	2.02
0.09	1.95	0.1	2.2	0.08	2.3	0.09	2.06	0.085	2.09	0.09	1.985
0.09	1.87	0.12	2.265	0.13	1.83	0.09	2.32	0.09	2.17	0.07	2.2
0.135	2.055	0.115	1.855	0.125	1.78	0.15	1.98	0.105	2.34	0.07	2.25
0.08	1.84	0.09	2.16	0.1	2	0.105	2.115	0.11	2.2	0.085	2.26
0.105	1.82	0.12	2.13	0.1	1.68	0.11	1.78	0.13	2.3	0.07	2.22
0.1	1.76	0.125	1.93	0.105	2.06	0.125	2.02	0.11	1.905	0.08	2.135
0.12	1.885	0.105	1.96	0.15	2.165	0.105	1.9	0.145	2.11	0.135	2.08
0.11	1.785	0.15	2.24	0.1	2.06	0.125	2.2	0.09	1.9	0.09	2.08
0.125	1.795	0.13	1.95	0.1	1.98	0.12	2.01	0.1	2.23	0.11	2.01
0.125	2.1	0.1	1.98	0.105	2.03	0.1	1.875	0.11	1.98	0.11	2.03
0.135	2.3	0.11	2	0.115	2.095	0.105	2.3	0.1	2.16	0.1	2.03

	Number of Offspring Hatched Per 3 Days					
Day 20	Day 3	Day 6	Day 9	Day 12	Day 15	Day 18
1	13	7	5	3	0	0
0	12	6	3	1	1	0
2	9	3	2	0	0	0
X	7	5	X	X	X	X
2	11	8	4	3	0	0
1	7	4	2	0	2	0
2	7	5	3	1	0	1
2	8	5	5	1	0	0
2	6	4	3	0	0	0
X	9	X	X	X	X	X
1	6	5	9	6	3	2
1	8	8	7	4	1	0
1	9	7	4	0	0	0
3	10	9	11	0	0	1
2	6	6	8	2	0	0
1	11	6	4	0	0	0
1	7	2	3	2	0	1
3	9	8	11	4	2	1
2	10	6	4	2	0	0
2	8	7	7	3	2	0
2	8	5	5	3	0	0
2	13	8	7	4	1	0
3	7	7	4	3	0	0
2	7	7	5	0	0	1
1	4	6	8	2	0	0
3	8	6	4	0	0	0
X	7	5	3	X	X	X
3	9	8	5	1	1	0
1	10	6	4	2	3	0
3	8	6	3	0	0	0
X	62	X	X	X	X	X
7	28	21	11	9	3	5
8	30	28	13	8	3	3
X	X	X	X	X	X	X
8	28	32	18	9	4	4
7	46	23	14	8	2	2
6	31	19	18	11	7	3
7	32	19	11	7	9	2

6	30	25	10	7	8	3
9	32	32	11	7	8	7
9	33	21	11	8	2	9
6	30	19	13	8	7	6
4	39	21	11	8	6	0
3	48	31	14	7	7	2
5	36	32	11	10	6	5
6	21	30	12	10	11	0
6	43	40	11	9	7	3
8	39	29	15	11	8	7
6	36	27	11	9	6	3
7	29	18	12	7	3	2

ne (in Days)											
**Day 57**	**Day 60**	**Day 63**	**Day 66**	**Day 69**	**Day 72**	**Day 75**	**Day 78**	**Day 81**	**Day 84**	**Day 87**	**Day 90**
64	60	51	51	40	32	26	22	18	13	4	0
0	1	0	2	2	0	0	0	0	1	1	0
64	59.5	51	50	39	32	26	22	18	12.5	3.5	0
4	8	0	9	6	6	4	4	5	8	3	0
0	0	0	0	0	0	0	0	0	0	0	0
0	0	0	0	0	0	0	0	0	0	0	0
0	0	0	0	0	0	0	0	0	0	0	0
0	0	0	0	0	0	0	0	0	0	0	0
25	21	18	14	12	10	6	4	3	2	0	0
0	0	1	0	0	2	0	0	0	0	0	0
25	21	17.5	14	12	9	6	4	3	2	0	0
4	3	3	2	2	2	2	1	1	2	0	0
14	11	10	5	5	3	3	3	0	0	0	0
0	0	1	0	0	0	0	0	0	0	0	0
14	11	9.5	5	5	3	3	3	0	0	0	0
3	1	4	0	2	0	0	3	0	0	0	0
16	16	11	8	8	7	5	4	3	3	1	1
0	0	0	0	0	1	0	0	0	0	0	0
16	16	11	8	8	6.5	5	4	3	3	1	1
0	5	3	0	1	1	1	1	0	2	0	1
9	9	5	3	0	0	0	0	0	0	0	0
0	2	0	0	0	0	0	0	0	0	0	0

9	8	5	3	0	0	0	0	0	0	0	0
0	2	2	3	0	0	0	0	0	0	0	0
33	32	25	23	19	13	10	9	7	2	0	0
0	3	0	0	1	0	0	0	0	0	0	0
33	30.5	25	23	18.5	13	10	9	7	2	0	0
1	4	2	4	5	3	1	2	5	2	0	0

Day 57	Day 60	Day 63	Day 66	Day 69
22	2	1	0	0
5	0	0	0	0
19.5	2	1	0	0
15	1	1	0	0
0	0	0	0	0
0	0	0	0	0
0	0	0	0	0
0	0	0	0	0
11	7	5	4	3
0	0	0	0	1
11	7	5	4	2.5
4	2	1	1	2
0	0	0	0	0
0	0	0	0	0
0	0	0	0	0
0	0	0	0	0
5	2	2	2	1
0	0	0	0	0
5	2	2	2	1
3	0	0	1	1
4	4	1	1	1
0	0	0	0	0
4	4	1	1	1
0	3	0	0	1
2	0	0	0	0
1	0	0	0	0
1.5	0	0	0	0
1	0	0	0	0

Day 30
11.5
9.5
9.5
9.5
9
9.5
8
10
9
11.5
11
10
10.5
13.5
9.5
9.5
10.5
9.5
12
10.5

y 45	Day 50	
Width	Length	Width
0.09	2.095	0.12
0.105	2.42	0.09
0.095	2.06	0.09
0.1	2.42	0.08
0.1	2.38	0.11
0.095	2.135	0.085
0.09	2.41	0.08
0.11	2.37	0.1
0.11	2.155	0.1
0.11	2.09	0.1

0.11	2.375	0.09
0.12	2.355	0.115
0.1	2.12	0.095
0.12	2.255	0.12
0.11	2.155	0.115
0.07	2.02	0.07
0.09	2.275	0.075
0.085	1.97	0.07
0.08	2.01	0.07
0.09	2.02	0.07
0.06	2.135	0.075
0.06	2.2	0.07
0.085	2.045	0.07
0.07	2.1	0.06
0.08	2.1	0.09
0.08	2.135	0.07
0.08	2.02	0.06
0.085	2.04	0.07
0.08	2.005	0.07
0.075	2.2	0.07
0.115	1.935	0.1
0.105	2.095	0.1
0.12	2.08	0.11
0.115	2.14	0.1
0.11	2.06	0.1
