# Supplementary material for: Men in menopause? Experimental verification of the mate choice theory with Drosophila melanogaster shows both sexes can undergo menopause
Source: PLoS One. 2025 Jul 3;20(7):e0326972. doi: 10.1371/journal.pone.0326972 (PMC12225806; doi:10.1371/journal.pone.0326972)
Supplement: S3 Table — (PDF) [file pone.0326972.s003.pdf]

Generation 70: Number of Eggs Lai					
C					
Experimental Group	Replicate	Vial #	Number of		
			Day 3	Day 6	Day 9
Old male-young female	1M	1	44	17	8
		2	61	7	3
		3	40	17	11
		4	42	16	6
		5	50	11	4
		6	42	12	6
		7	54	24	6
		8	46	1	0
		9	49	2	1
		10	38	6	5
		11	62	14	7
		12	52	8	11
		13	40	11	9
		14	56	16	0
	2M	1	33	20	7
		2	61	15	0
		3	60	20	6
		4	58	13	0
		5	60	20	16
		6	59	17	4
		7	58	22	5
		8	60	24	9
		9	62	23	0
		10	66	17	7
		11	46	16	6
		12	49	19	10
	3M	1	59	18	22
		2	39	2	1
		3	21	9	12
		4	48	2	3
		5	41	27	1
		6	62	17	12
		7	61	19	13
		8	38	12	10
		9	46	10	4
		10	69	16	20

		**11**	51	14	11
		**12**	58	17	9
Control	Control	**1**	61	30	21
		**2**	67	17	8
		**3**	53	30	16
		**4**	63	36	11
		**5**	61	25	9
		**6**	72	36	16
		**7**	78	32	12
		**8**	55	31	14
		**9**	61	42	21
		**10**	57	25	15
		**11**	65	35	16
		**12**	56	28	18
		**13**	82	34	16
		**14**	59	43	20

**Old female-young male**						
**Experimental Group**	**Replicate**	**Sub-group**	**Vial #**	**Number of Eggs Laid**		
				**Day 3**	**Day 6**	**Day 9**
		Males aged 3 days pre-mating	**1**	43	25	4
			**2**	54	30	10
			**3**	71	7	3
			**4**	76	7	0
			**5**	58	19	11
			**6**	59	21	6
			**7**	62	23	4
			**8**	53	30	6
			**9**	43	28	15
			**10**	39	29	17
			**11**	63	20	12
			**12**	68	15	10
			**13**	57	22	16
			**14**	51	33	11
			**15**	54	23	15
			**1**	28	14	4
			**2**	33	26	1
			**3**	24	11	8
			**4**	37	12	2
			**5**	26	18	2
			6	42	15	4

	1F	Males aged 6 days pre-mating	7	27	15	9
			8	35	12	0
			9	49	10	9
			10	35	11	0
			11	37	8	2
			12	27	9	3
			13	36	12	0
			14	29	16	0
			15	36	11	3
		Males aged 9 days pre-mating	1	9	9	3
			2	7	3	4
			3	12	2	0
			4	16	5	5
			5	9	4	0
			6	8	0	0
			7	12	8	1
			8	7	2	2
			9	6	3	0
			10	14	1	8
			11	8	1	1
			12	1	7	4
			13	10	0	0
			14	3	3	1
			15	11	0	0
		Males aged 3 days pre-mating	1	41	29	12
			2	55	22	10
			3	56	25	12
			4	68	14	0
			5	78	19	1
			6	42	29	8
			7	65	30	3
			8	78	14	2
			9	47	29	5
			10	58	31	1
			11	55	28	14
			12	62	22	3
			13	39	28	13
			14	55	40	1
			15	57	33	9
			1	43	6	0
			2	28	12	8

Old female-young male	2F	Males aged 6 days pre-mating	3	38	8	1
			4	37	11	1
			5	35	16	2
			6	37	15	0
			7	43	4	0
			8	42	6	6
			9	36	15	7
			10	38	3	0
			11	25	8	1
			12	37	6	0
			13	41	19	1
			14	53	5	3
			15	41	9	9
		Males aged 9 days pre-mating	1	10	3	0
			2	11	9	1
			3	13	5	4
			4	2	20	0
			5	7	2	0
			6	12	1	4
			7	14	3	1
			8	1	5	0
			9	11	1	0
			10	3	3	5
			11	5	2	0
			12	8	4	3
			13	18	7	2
			14	12	9	0
			15	6	6	0
		Males aged 3 days pre-mating	1	56	19	5
			2	47	26	9
			3	59	22	10
			4	53	21	16
			5	61	23	12
			6	56	20	8
			7	49	21	5
			8	55	22	7
			9	57	18	9
			10	49	19	0
			11	52	29	7
			12	61	24	9
			13	56	23	15

			14	45	31	4
			15	52	28	3
	3F	Males aged 6 days pre-mating	1	22	11	6
			2	34	12	2
			3	31	19	4
			4	33	15	3
			5	29	14	0
			6	22	15	7
			7	45	10	3
			8	27	9	2
			9	34	14	4
			10	33	13	1
			11	29	13	2
			12	35	15	8
			13	41	12	0
			14	28	11	0
			15	41	14	5
		Males aged 9 days pre-mating	1	19	2	1
			2	14	9	0
			3	15	2	0
			4	6	3	0
			5	3	0	0
			6	11	6	0
			7	14	0	1
			8	9	5	1
			9	10	7	0
			10	6	1	1
			11	12	1	0
			12	0	1	0
			13	5	2	1
			14	8	6	1
			15	4	0	0
		Males aged 3 days pre-mating	1	70	24	12
			2	73	27	6
			3	65	21	14
			4	58	22	21
			5	61	28	18
			6	53	35	15
			7	55	38	17
			8	65	37	7
			9	52	24	19

Control	Control		10	55	32	12
			11	51	28	20
			12	73	21	14
			13	58	26	13
			14	62	29	15
			15	71	30	8
		Males aged 6 days pre-mating	1	51	14	3
			2	53	13	5
			3	62	20	6
			4	44	17	2
			5	59	12	4
			6	53	26	1
			7	46	16	7
			8	53	15	2
			9	46	19	8
			10	51	24	3
			11	52	13	2
			12	46	27	3
			13	55	24	9
			14	47	14	2
			15	48	18	4
		Males aged 9 days pre-mating	1	11	9	7
			2	11	6	2
			3	15	13	1
			4	18	9	7
			5	17	9	6
			6	9	8	8
			7	6	5	1
			8	12	7	5
			9	13	1	0
			10	13	3	1
			11	20	3	1
			12	13	4	4
			13	11	8	2
			14	9	5	4
			15	17	0	0

d and Offspring Hatched by Experimental Group per Day

)ld male-young female

f Eggs Laid				Number of Offspring Hatche		
Day 12	Day 15	Day 18	Day 3	Day 6	Day 9	Day 12
4	3	1	28	17	2	2
0	0	0	39	7	0	0
4	1	1	33	17	2	0
1	0	4	39	5	5	0
2	0	0	30	5	1	0
4	1	1	27	3	1	3
2	0	0	32	18	4	2
0	0	0	30	0	0	0
0	0	0	24	0	0	0
0	0	1	32	0	0	0
6	2	0	36	8	7	5
7	3	3	35	7	6	1
6	1	4	28	7	7	2
9	8	2	29	12	0	0
5	4	3	15	18	7	3
0	0	0	35	10	0	0
3	0	0	32	13	3	0
3	1	2	33	11	0	1
8	1	1	30	6	0	0
2	4	1	33	12	2	1
2	4	2	24	13	3	2
1	1	3	37	13	4	0
1	0	0	43	5	0	0
10	2	3	25	8	3	7
6	5	6	31	4	2	0
8	1	0	32	15	9	0
23	2	1	30	9	3	0
0	0	0	6	1	0	0
4	1	1	14	1	0	0
2	5	4	34	2	1	0
11	1	5	30	12	0	0
9	2	1	34	11	5	1
5	8	4	53	10	7	3
3	0	4	28	9	8	0
0	0	0	31	10	3	0
12	5	4	38	15	18	2

7	2	0	30	13	5	0
2	0	3	0	9	7	2
0	4	3	47	20	14	0
1	0	0	40	14	6	1
1	6	4	42	26	14	1
9	1	0	42	28	5	8
6	5	2	43	22	6	6
7	4	2	44	33	14	2
7	1	1	61	16	9	0
4	6	5	42	30	8	0
8	3	5	42	39	19	8
7	10	4	39	18	12	1
4	8	4	41	31	14	4
9	6	4	31	17	15	7
13	4	6	69	24	9	6
15	8	11	45	26	13	7

Number of Eggs Hatched		
**Day 3**	**Day 6**	**Day 9**
39	18	0
55	17	4
63	4	2
59	0	0
37	15	6
52	16	5
45	21	7
55	18	2
39	17	12
28	19	12
52	18	7
45	13	9
50	14	6
41	20	5
46	16	7
16	6	4
27	15	1
24	0	5
25	12	1
22	16	1
37	9	0

25	11	4
33	6	1
43	9	0
25	4	0
30	5	1
25	3	0
26	1	0
18	16	0
29	7	3
2	2	3
3	1	3
4	1	0
9	5	4
0	0	0
0	0	0
6	1	0
6	1	0
1	0	0
12	2	1
8	1	0
0	3	2
9	0	0
3	1	1
8	0	0
37	18	9
53	15	7
43	19	11
56	4	0
61	15	0
35	21	3
50	19	1
52	9	0
55	25	2
54	11	0
46	23	7
59	19	0
31	22	8
48	17	0
48	24	4
38	0	0
24	10	2

29	5	5
26	5	2
33	9	0
35	9	1
40	1	0
39	5	1
31	8	1
26	0	0
22	0	0
34	0	0
35	11	0
49	2	0
39	2	1
8	0	0
5	6	0
9	2	2
0	0	0
7	0	0
10	1	3
9	0	0
0	0	0
4	1	0
2	3	4
4	1	0
8	4	2
12	3	1
7	6	0
2	2	0
32	9	4
38	18	0
48	14	10
42	7	11
54	8	0
52	18	8
40	10	5
52	9	5
50	11	0
38	17	0
41	12	6
43	16	1
39	17	11

38	21	0
42	18	1
18	7	5
21	9	0
21	10	4
19	12	0
24	12	0
21	3	2
33	0	0
23	8	0
29	11	2
18	3	1
22	12	0
26	13	8
39	10	0
13	8	0
25	5	4
7	2	1
5	2	0
5	1	0
4	3	0
0	0	0
1	0	0
0	0	0
2	0	0
3	4	0
5	1	1
0	1	0
0	0	0
1	1	1
4	2	0
3	0	0
68	8	10
73	15	6
60	15	7
47	19	14
52	20	12
55	33	5
46	35	10
34	37	4
43	20	11

43	31	6
52	19	13
66	17	10
48	25	6
54	27	12
53	28	8
47	14	2
53	12	5
54	15	4
40	15	1
53	8	3
44	18	1
41	15	6
50	11	2
46	6	3
35	20	3
37	11	2
28	21	1
48	22	6
43	9	2
39	16	1
9	7	5
11	5	1
10	12	1
17	6	3
16	5	3
6	5	4
4	3	1
9	2	1
5	1	0
12	2	1
17	1	1
7	3	2
10	8	2
5	5	3
15	0	0

d Day 15	Day 18
2	0
0	0
0	0
0	0
0	0
1	1
0	0
0	0
0	0
0	1
1	0
1	0
0	3
0	0
2	3
0	0
0	0
0	1
0	0
3	0
1	0
0	1
0	0
0	0
1	2
1	0
0	0
0	0
0	0
2	0
0	0
0	0
3	1
0	0
0	0
1	0

0	0
0	1
3	3
0	0
6	3
1	0
4	2
2	1
0	1
5	5
3	5
4	1
4	1
1	1
3	1
4	3
